# Nanotherapeutic Approaches to Treat COVID-19-Induced Pulmonary Fibrosis

**DOI:** 10.3390/biotech12020034

**Published:** 2023-05-05

**Authors:** Shrey Kanvinde, Suyash Deodhar, Tanmay A. Kulkarni, Chinmay M. Jogdeo

**Affiliations:** 1 Department of Pharmaceutical Sciences, University of Nebraska Medical Center, Omaha, NE 68198, USA; 2 Department of Pharmacology and Experimental Neuroscience, University of Nebraska Medical Center, Omaha, NE 68198, USA

**Keywords:** SARS-CoV-2, COVID-19, pulmonary fibrosis, coronavirus, nanoparticles, nanomedicine, pandemic, acute respiratory syndrome

## Abstract

There have been significant collaborative efforts over the past three years to develop therapies against COVID-19. During this journey, there has also been a lot of focus on understanding at-risk groups of patients who either have pre-existing conditions or have developed concomitant health conditions due to the impact of COVID-19 on the immune system. There was a high incidence of COVID-19-induced pulmonary fibrosis (PF) observed in patients. PF can cause significant morbidity and long-term disability and lead to death in the long run. Additionally, being a progressive disease, PF can also impact the patient for a long time after COVID infection and affect the overall quality of life. Although current therapies are being used as the mainstay for treating PF, there is no therapy specifically for COVID-induced PF. As observed in the treatment of other diseases, nanomedicine can show significant promise in overcoming the limitations of current anti-PF therapies. In this review, we summarize the efforts reported by various groups to develop nanomedicine therapeutics to treat COVID-induced PF. These therapies can potentially offer benefits in terms of targeted drug delivery to lungs, reduced toxicity, and ease of administration. Some of the nanotherapeutic approaches may provide benefits in terms of reduced immunogenicity owing to the tailored biological composition of the carrier as per the patient needs. In this review, we discuss cellular membrane-based nanodecoys, extracellular vesicles such as exosomes, and other nanoparticle-based approaches for potential treatment of COVID-induced PF.

## 1. Background

Pulmonary fibrosis (PF) is a disease characterized by decline in lung function, which eventually leads to respiratory failure if untreated. PF is a progressive disease with lung transplant being the only option in late stages [1]. In some cases, PF has histopathologic and radiologic indications that are like other non-PF forms of interstitial lung disease, making it difficult to distinguish for accurate diagnosis [2]. Histologically, it is characterized by permanent scarring and extracellular matrix deposition in lung parenchyma [3,4]. Recent advances have determined certain genomic as well as natural history factors that increase the risk of PF. It has been reported that a single nucleotide polymorphism on the p-terminus of chromosome 11, which is located within a highly conserved area of the promoter region for the mucin 5B (MUC5B) gene, is associated with the high risk of PF [5]. Other polymorphisms and loci have also been identified that are associated with high risk of PF. Interestingly, members of extended families with variant abnormalities of the same gene were at high risk of different fibrotic forms of lung injury [6]. Genomic factors and epigenetic mechanisms such as histone modification and microRNA expression are also found to be responsible for high risk of fibrosis [7,8]. Moreover, advanced age is likely to increase the risk of PF, males are at higher risk compared to females, and occupational hazards such as silica exposure, material dust exposure, and livestock exposure have been shown to contribute to the pathogenesis of PF as well [9,10]. However, it should be noted that in many cases, patients with PF do not necessarily have a prevalence of the previously mentioned risk factors; however, these risk factors do require genetic predisposition to develop PF. The key histologic indications of PF are characteristic fibroblastic foci accompanied by distortion and fibrosis of lung tissue. Primary symptoms of PF include persistent dry cough, shortness of breath, tiredness, and weight loss. If undiagnosed, the disease may worsen, causing pneumonia or bronchitis followed by chest pain and cardiac failure in the later stages of disease progression. According to the World Health Organization (WHO), about 100,000 people are affected by PF in the United States, and 30,000 to 40,000 new cases are diagnosed each year. Moreover, the global prevalence of PF is 13 to 20 cases per 100,000 people.

In the post-COVID-19 era, PF is also viewed as an aftereffect of COVID-19 infection. PF was not only a major cause of death worldwide in COVID-19 infected patients but also a cause of permanent lung tissue damage in recovered patients [11,12]. COVID-19 and PF also share common risk factors such as old age, male sex, and comorbidities such as diabetes and hypertension. Current treatment options for PF include antifibrotic agents such as pirfenidone and nintedanib. Although these therapies are shown to improve survival expectancy by as much as 2 years, they have been effective in controlling the rate of lung function decline by only about 50% [13,14,15]. Additionally, these therapies are available only in the form of oral dosage forms, and hence, administration is difficult for patients in coma or patients that are intubated/mechanically ventilated in critical care units. 

Nanotherapeutic approaches, by virtue of their physical and chemical characteristics such as size, charge, chemical composition, and surface modification, can provide advantages in terms of administration ease as well as therapeutic efficacy. Their small size allows for administration by parenteral routes, which is a significant advantage for intubated patients. Owing to their nanoscale size, nanoparticles can effectively deliver the drug cargo to the target site with minimal exposure to healthy tissues and organs. As a result, drug dumping is avoided, and the required dose can be reduced. This is particularly important in the case of potent drugs and steroids. The small size of nanoparticles and other nanotherapeutics results in a net large surface area, and the external surface of nanotherapeutics can be modified with ligands that bind to specific cell receptors. This allows for drug delivery at the cellular level within organs. The biological and chemical composition of the nanotherapeutics can be modified to allow for drug release upon specific stimuli present within the target cell. Such strategies further ensure that maximal drug is available only upon cellular internalization. Nanotherapies offer advantages in the case of combination therapy of drugs with varying pharmacokinetic characteristics. Packaging in a nanoparticle facilitates the availability of multiple drugs at the target site at the same time, hence achieving “true” combinatory therapeutic effects.

In this review, we discuss various nanotherapeutic approaches that can potentially be used in the control, maintenance, and treatment of PF.

## 2. Pathophysiology of COVID-19-Induced PF

**Figure 1:**
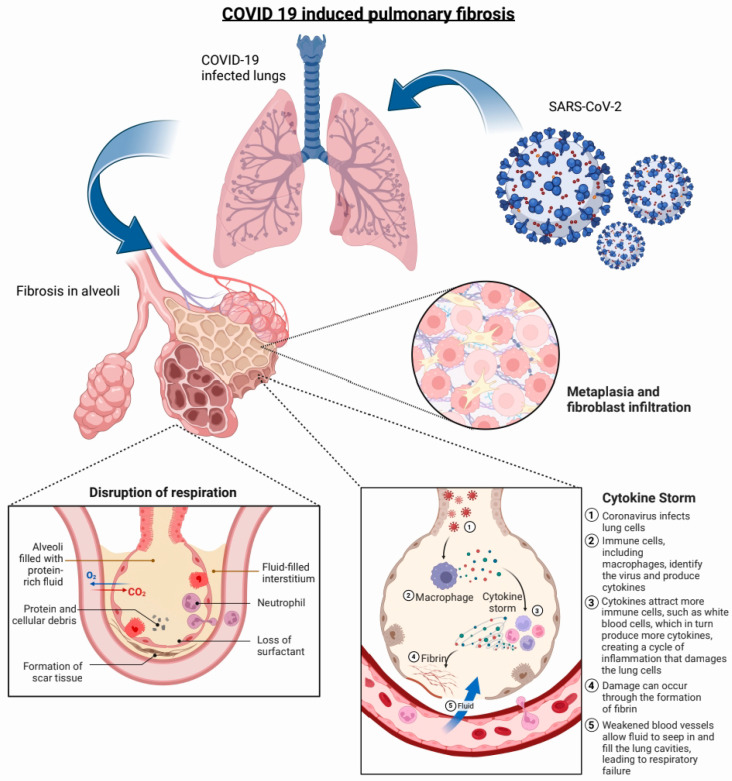
Pathophysiology of COVID-induced PF. The aberrant interaction of severe acute respiratory syndrome coronavirus-2 (SARS-CoV-2) with the alveolar epithelium (Type I and II) leads to cellular death, inflammation, and keratinization. Disruption of respiration is a grave cardinal symptom of PF and has been linked to patients affected severely by COVID-19. The molecular mechanisms behind the positive feedback loop of inflammatory activity have been linked to a phenomenon of “cytokine storm” mediated through monocytes and myeloid cells. A more complex mechanism of cellular metaplasia of krt+ basal cells and fibroblasts to the alveoli has been linked to decreased lung function, the furtherance of aberrant inflammatory responses, and reduced overall survival. (Created with BioRender.com.)

While the complex molecular mechanisms that precipitate the grave pathophysiological outcomes in the lung are being elucidated, a significant extent of overlap in the progression and genetic etiology of idiopathic pulmonary fibrosis (IPF) and COVID-19-induced PF has been demonstrated [16,17]. The heterogeneity in the severity of the disease progression for COVID-19 has led to a wide spectrum of health outcomes, ranging from asymptomatic to needing mechanical ventilation, lung damage, and death [18]. The downstream pulmonary effects produced by COVID-19 overlap uniquely with the signaling pathways shown by IPF. Figure 1 shows a representation of the pathophysiology of COVID-19-induced PF. The macrophage infiltration, progressive fibroblast proliferation, and metaplasia have been reported in patients with COVID-19, while not correlated with H1N1 influenza or bacterial infections [19,20]. In approximately 84% of COVID-19 patients, signs and symptoms of PF follow a clinical cure and are seen in all patients with severe or critical SARS-CoV-2 infection [12]. Fadista and co-workers reported a positive genetic correlation of IPF and the severity of COVID-19 symptoms in an age-stratified Mendelian randomization analysis. However, there is also parallel evidence signifying a divergence in the etiology of PF caused by COVID-19 and IPF. Flaifel and co-workers reported the incidence of a non-specific interstitial pneumonia (NSIP) and bronchiectasis in COVID-19 patients instead of the interstitial pneumonia pattern of fibrosis seen in IPF, and this finding was also corroborated by Parimon and co-workers [21].

A key element of the etiology of COVID-19-induced PF is the preferential infectivity that this virus has toward alveolar macrophages and the basal epithelial cells in the respiratory tract, owing to the attachment protein ACE2 [22]. The alveolar epithelial cells (AECs) play a key role in pulmonary homeostasis, with Type I AECs regulating gas exchange, while the Type II AECs are responsible for the production of a protein–lipid surfactant containing surfactant protein C, sterols, and dipalmitoylphosphatidylcholine (DPPC), which is critical for lowering of the surface tension and alveolar function [23,24]. The epithelial–mesenchymal trophic units (EMTUs) are a functional element representing the feedback interactions between the epithelial and mesenchymal cells [25,26,27]. This signaling channel can be disrupted by cellular death, disruption, and inflammation in the type II AECs perpetuated by environmental factors such as silica or metal dust, radiation, or infections such as COVID-19. The surviving type II AECs have been shown to overexpress profibrotic markers such as pSTAT3 and KIT, apoptotic markers such as CASP3, and proinflammatory signaling molecules such as IL-6.

The persistence of inflammation in these loci has direct links to increased levels of proinflammatory biomarkers such as IFN-γ type 2, TGF-β, IL-1α, and IL-8 [28,29]. Monocytes, macrophages, and myeloid cells induced by these signaling markers further trigger a “cytokine storm” and thus perpetuate a positive feedback loop with further immune cell recruitment, ROS generation, and colocalization with lung tissue showing profibrotic histology [30,31]. The high degree of chronic inflammation found in COVID-19 patients with PF remains a key feature of the condition [32]. Aberrant activation of lung epithelial progenitor cells that leads to increased active myofibroblasts with associated fibroblast alveolar migration is a downstream effect of the disrupted cell signaling in PF. Indeed, proliferation of myofibroblasts with other elements of the mesenchyme has been reported in patients with late- or end-stage fibrotic COVID-19 [33]. Alveolar epithelial damage and the subsequent dysregulation of the paracrine cell signaling contribute to the aberrant cell proliferation that leads to metaplasia, a critical feature of PF [34,35,36,37]. The altered numbers and function of the type II AECs lead to metaplastic infiltration of krt+ basal cells into the alveoli, subsequent keratinization of the pulmonary tissue, and eventually decreased lung function [38,39,40]. This reduced lung function, overexpressed TGF-β signaling, and progression of PF are linked to decreased overall survival rates [41]. TGF-β plays a key role in the progression of PF by inducing recruitment of myofibroblasts, accumulation of extracellular matrix, and persistent induction of fibronectin and collagen [42,43,44]. The effects of TGF-β associated cascades are balanced by the activity of the bone morphogenetic protein (BMP-4), which is responsible for inhibiting the proliferation of human pulmonary fibroblasts and induces the proliferation of pulmonary epithelial cells [45]. Koli and co-workers demonstrated elevated levels of Gremlin/Drm mRNA, an antagonist of BMP-4, in fibrotic tissue [42]. Tilting of the balance toward TGF-β cascades in antagonism to BMP-4 in fibrotic tissue has been demonstrated in murine models. There might be a diagnostically relevant unique expression profile for PF marked by lower levels of circulating IFN-β and higher IL-1α and TGF-β. 

**Figure 2:**
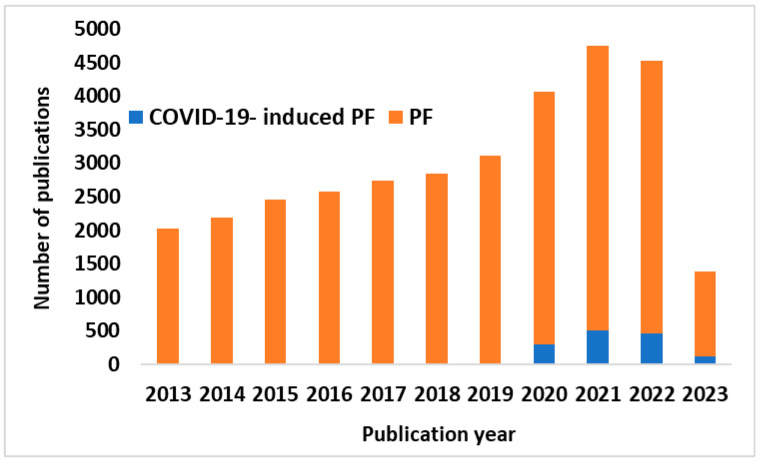
Number of publications on PF vs. COVID-19-induced PF. Results of a PubMed search over the last 10 years (as of 10 April 2023) for the terms “COVID-19-induced PF” (*blue*) and “PF” (*orange*).

The overall pathophysiology of PF and the high extent of overlap with COVID-19 warrant further investigation into the metabolome and the interactome of fibroproliferative cell signaling, metaplasia, and immune activity. The covariables of history of exposure to tobacco smoke, environmental irritants, chronic obstructive pulmonary disorder (COPD), autoimmunity, as well as genetic predisposition may also contribute to the severity of the PF seen in COVID-19 patients. This attention to PF as an effect of COVID-19 has led to a proliferation of reports on COVID-19-induced PF. This is clearly observed in literature searches using the terms “PF” and “COVID-19-induced PF”. A simple PubMed search utilizing these terms resulted in nearly 1400 hits (Figure 2). If there is so much realization about this topic, however, then why are there comparatively so few studies looking at delivery of therapeutics that take COVID-19-induced PF into consideration? Thus, the main goal of this review article was to discuss the status of COVID-19-induced PF therapies and provide information on reported nanotherapeutic approaches that may yield more promising results in the future.

## 3. Current Status of COVID-19-Induced PF Therapy 

Current therapy of PF is performed with either pirfenidone or nintedanib. Pirfenidone’s mechanism of action is not completely understood yet; however, it is believed to inhibit fibroblast activity and matrix deposition by disrupting TGF-β activity [46]. Pirfenidone underwent multiple clinical trials; however, three separate sets of trials led to its authorization for use in Japan, Europe, and the USA [47]. A randomized, double-blind, placebo-controlled phase III trial that included mild-to-moderate PF patients was conducted in Japan over 52 weeks [48]. The progression-free survival was significantly longer in the 1800 mg/day dose arm compared to the placebo arm, leading to authorization in Japan. Moreover, two CAPACITY trials were conducted by enrolling mild-to-moderate progression PF patients, of which one trial, i.e., trial 004, met the primary endpoint [49]. Trial 006, on the other hand, could not demonstrate a difference between pirfenidone-treated subjects as compared to placebo arm in terms of the % change in forced vital capacity (FVC) of the lungs. These trials earned pirfenidone authorization for use in Europe. However, an additional study was required by the US FDA. 

The ASCEND study enrolled patients with high risk of disease progression [15]. This trial demonstrated a 45% reduction in FVC decline compared to placebo arm, satisfying the primary objective of this trial. Following this study, the FDA granted pirfenidone an authorization for use in PF. The reported side effects of pirfenidone include nausea, vomiting, dyspepsia, anorexia, photosensitivity reaction, skin rash, and elevation of liver enzymes. Pirfenidone is metabolized primarily in the liver via the cytochrome system (CYP1A2 enzyme). Hence, co-administration of CYP1A2 inhibitors (e.g., amiodarone, ciprofloxacin, fluvoxamine) or inducers (e.g., omeprazole) that can alter pirfenidone bioavailability is avoided [50,51]. Pirfenidone is contraindicated in patients with severe hepatic dysfunction or patients with advanced renal dysfunction (glomerular filtration rate < 30 mL/min). On the other hand, nintedanib inhibits multiple intracellular kinases by blocking receptors for vascular endothelial growth factor (VEGFR 1–3), platelet-derived growth factor (PDGFR α and β), and fibroblast growth factor (FGFR 1–3), and it also inhibits Fms-like tyrosine kinase-3. In INPULSIS-1 and -2 trials, it showed reduced decline in FVC in nintedanib-treated patients as compared to placebo arm [14]. The most common side effects are diarrhea and gastrointestinal discomfort. Nintedanib is mainly metabolized by hepatic ester cleavage, and a minor role is played by CYP3A4. It is also a substrate for p-glycoprotein. Hence, co-administration of inhibitors of both CYP3A4 and P-glycoprotein (e.g., erythromycin, imidazoles) or inducers (e.g., phenytoin, carbamazepine, rifampicin, St. John’s Wort) is avoided. Additionally, nintedanib is contraindicated in patients with moderate to severe hepatic dysfunction. Additionally, both these drugs hold potential to be explored for their use in COVID-19-induced PF. However, clinical studies to determine whether these drugs help prevent post-COVID fibrotic injury have not taken place yet, apart from the few case studies in combination with other drugs. Additionally, safety and specificity to lung tissue remain viable concerns for all the currently utilized therapies. Table 1 shows ongoing clinical trials to develop therapies against COVID-19-induced PF. 

As widely reported for other disease areas, nanomedicine can overcome the limitations of the current PF therapies [52,53]. Nanotherapies such as nanoparticles, cell-derived vesicles, and nanopolymers are more amenable to modification to enhance their safety, specificity, and stimuli-responsive drug release [54,55]. Additionally, there have been multiple reports of tailoring the pharmacokinetics of drugs by using nanomedicine-based approaches [56]. Nanoparticle applications to deliver drug combinations have been widely reported [57,58]. To summarize efforts, specifically looking at the applications of nanotherapeutic approaches to COVID-19-induced PF, an exhaustive search on PubMed was performed. Articles reported from 2020 were screened to filter out articles that reported approaches to treat PF and focus on articles reporting COVID-19-induced PF. Research articles that described nanotherapeutic approaches were selected, and specific emphasis was put on reports that included in vivo data. In the following section, we discuss a few nanotherapeutic approaches reported in the literature that look specifically at COVID-19-induced PF.

## 4. Nanotherapeutic Approaches to Treat COVID-19-Induced PF

**Figure 3:**
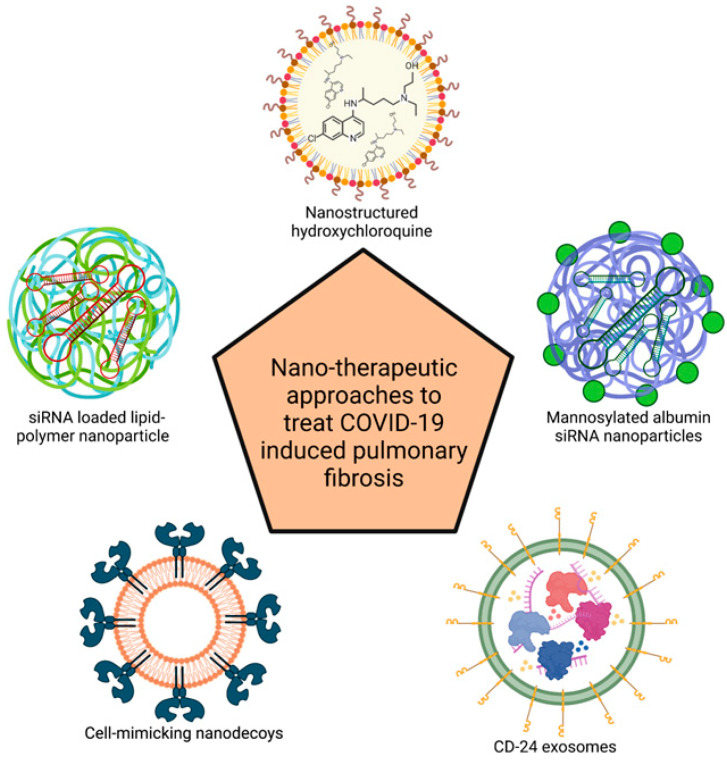
Graphical representation of currently reported delivery systems explored in treatment of COVID-19-induced PF. (Created with BioRender.com).

This section focuses on different types of nanotherapeutic approaches that have been reported in literature. Figure 3 depicts graphical representation of these reported nanotherapeutic approaches.

### 4.1. Cell-Mimicking Nanodecoys

Reports that elucidated the mechanism of COVID-19 indicated that angiotensin-converting enzyme 2 (ACE2) plays a pivotal role in viral entry into the host cell [59,60]. The role of ACE2 has been well characterized in multiple viral infections. In COVID-19, the spike protein of the virus specifically interacts with ACE2-presenting pneumocytes in the lungs and goblet secretory cells in the nasal mucosa [61]. Li and co-workers developed a cell therapy using a mixture of resident lung epithelial cells and mesenchymal cells that they termed lung spheroid cells (LSCs) [62]. Since these cells express ACE2, the authors utilized their cell membrane to fabricate ACE2 nanodecoys. The LSC-nanodecoys could act as cell mimics and bind to the SARS-CoV-2 spike (S) protein, consequently triggering a phagocytotic response from macrophages resulting in the elimination of the virus. This therapeutic system provided an advantage over spike protein targeting therapies such as antiviral drugs and vaccines, as they target human cells expressing the ACE2 receptor and are independent of viral proteins. Consequently, the authors are of the view that this therapy could also be more effective in managing more aggressive variants associated with the mutations in the viral spike proteins.

**Figure 4:**
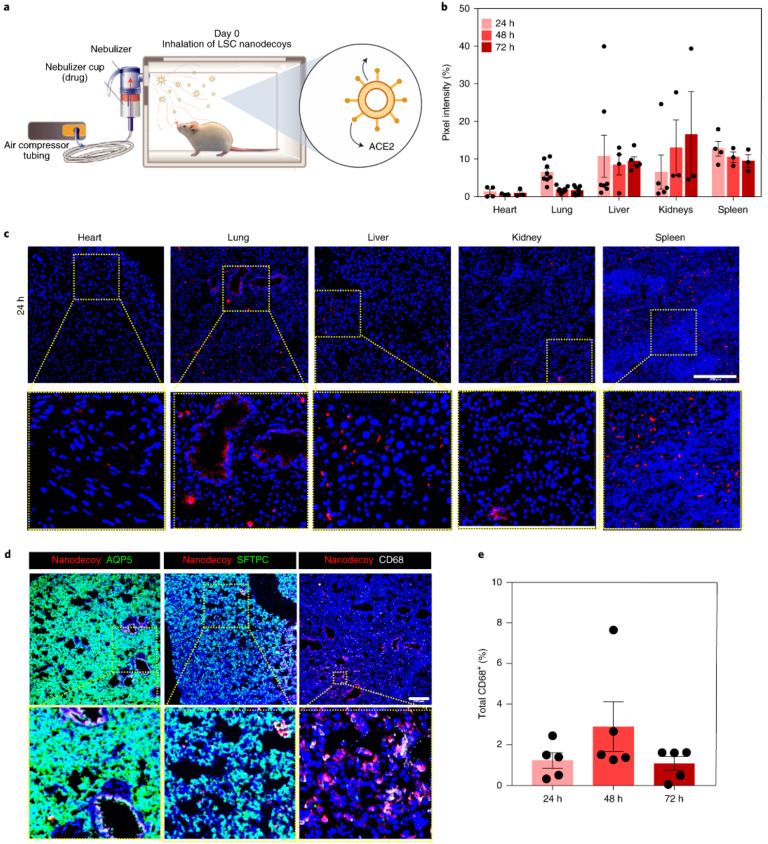
Biodistribution of nanodecoys in CD1 mice on inhalation. (**a**) Schematic demonstrating study design. (**b**) Quantitative results showing biodistribution in various organs using fluorescent labeled nanodecoys. (**c**) Confocal images showing fluorescent nanodecoys (red) in tissue sections. (**d**) Confocal images showing co-localization of nanodecoys with lung cells and macrophages. (**e**) Percentage of nanodecoy-positive macrophages. Reprinted with permission from Li Z. et al. Nature Nanotechnology volume 16, pages 942–951 (2021) [62]. Copyright 2021 Nature Publishing Group.

Nanodecoys were generated by serial extrusion of LSCs through polycarbonate membranes with various pore sizes and characterized. The obtained nanodecoys exhibited an average size of 320 nm and were spherical in shape. A yield of approximately 11,000 nanodecoys was obtained from one LSC. Control nanodecoys were also generated from HEK293 cells, which do not express the ACE2 receptors. The authors also demonstrated the ability of nanodecoys to bind to S-protein in a dose-responsive manner. While LSC nanodecoys could competitively bind within 4 h, HEK293 nanodecoys did not show binding to S-protein. In addition, LSC-nanodecoys were internalized more in the macrophage cells as compared to lung cells, indicating that they could be potentially cleared out by the immune cells. Similar studies were also performed to show that the LSC nanodecoys could protect from SARS-CoV2 mimics. The biodistribution of the nanodecoys was performed in CD1 mice (Figure 4). Fluorescent labeled nanodecoys were administered to mice via inhalation route, and biodistribution analysis was performed at 24, 48 and 72 h post administration. The authors reported that nanodecoys could still be found in the lungs 72 h after a single inhalation treatment. In addition, nanodecoys were also found in spleen, liver and kidney, indicating clearance via the reticuloendothelial system (RES). No impact was observed on CD68+ macrophage infiltration, indicating biocompatibility with immune cells. Additionally, the authors also demonstrated that the LSC nanodecoys could significantly reduce the number of SARS-CoV2 mimics that were internalized by lung cells, avoiding further infection. This effect was not observed for the free form of recombinant ACE2 or HEK293 nanodecoys. Confocal microscopy assessments showed that the nanodecoys could accelerate the clearance of the mimics. Cytokine and histopathological assessments of all major organs showed no adverse effects of either LSC or control nanodecoys.

**Figure 5:**
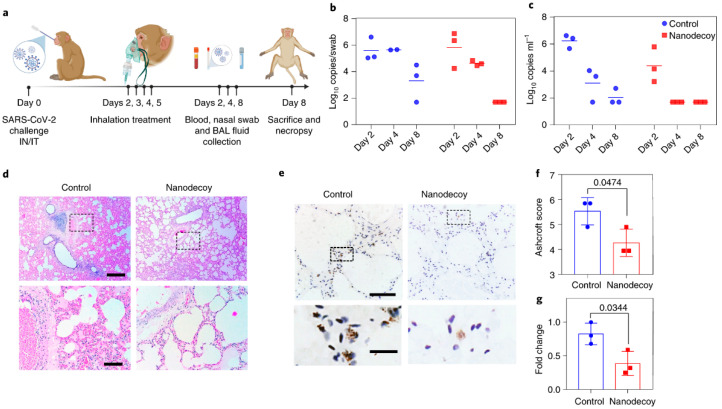
Treatment of SARS-CoV-2 infection in cynomolgus macaques. (**a**) Schematic demonstrating study design. (**b**,**c**) Quantification of viral load in NS and BAL on treatment. (**d**,**e**) Representative histological images of lung tissue stained with H&E and IHC staining. (**f**) Quantification of lung fibrosis using Ashcroft scoring. (**g**) Quantification of positive SARS-N numbers in lung tissues. Reprinted with permission from Li Z. et al. Nature Nanotechnology volume 16, pages 942–951 (2021) [62]. Copyright 2021 Nature Publishing Group.

To demonstrate therapeutic efficacy of the LSC nanodecoys, a pilot non-human primate (NHP) study was performed in cynomolgus macaques (Figure 5). This model could replicate many clinical symptoms of SARS-CoV-2 infection as well as show robust viral replication. The animals were challenged with SARS-CoV-2 using intranasal and intrathecal routes followed by random assignment into the LSC nanodecoy and PBS control groups. Nanodecoys were administered daily from days 2 to 5 post-challenge via inhalation using a nebulizer and fitted mask at the dose of 10^10^ particles/kg (Figure 5a). Eight days post-challenge, the animals were sacrificed for further assessments. Viral loads in bronchoalveolar lavage (BAL) and nasal swabs (NS) were assessed by using RT-PCR to measure genomic RNA that was indicative of viral replication (Figure 5b,c). High levels of RNA were observed in control animals, with a median peak of 6.243 log_10_RNA copies mL^−1^ in BAL and a median peak of 5.595 log_10_RNA copies per swab in NS on day 2. Contrarily, RNA levels showed a dramatic reduction in nanodecoy-treated animals, with a <1.7 log_10_ reduction of median peak RNA in both BAL and NS on day 8 post-challenge. Histopathological analysis of the lung tissue was performed at the end of the study (Figure 5d,e). Multifocal regions of inflammation, monocellular cell infiltrates and edema were observed in the control animals. On the other hand, LSC-nanodecoy treatment significantly reduced the numbers of inflammatory cells infiltrating the lungs. Immunohistochemistry (IHC) staining was performed to detect the viral levels within the lung tissue. The LSC-nanodecoy treated group showed significantly lower levels of viral protein as well as viral replication within the lung tissue as compared to control groups.

Based on this study, the authors were able to show that the nanodecoys not only showed specificity towards lung accumulation but also exerted their effect only in the impacted tissue without eliciting toxicity in other systemic organs. Based on all of these findings, the authors believe that LSC-nanodecoys can serve as a potential therapeutic agent for treating COVID-19 and COVID-19-induced PF. However, the authors mentioned that in terms of drug development, the major pain points they wanted to address were off-target effects and undesired biodistribution. Similarly, several challenges including process development to ensure large-scale production of LSCs, development of a robust manufacturing protocol and establishment of updated regulatory guidelines need to be addressed to ensure clinical translation of these nanodecoys. 

### 4.2. CD-24 Exosomes

Shapira and co-workers tried to develop a therapy based on CD24, which is a small, heavily glycosylated membrane-anchored protein that acts as an immune checkpoint regulator [63]. CD24 allows immune differentiation between molecular patterns of damaged or dying cells versus the ones derived from pathogens such as bacteria and viruses [64]. Binding of CD23 to damage associated molecular patterns (DAMPs) prevents binding to pathogen associated molecular patterns (PAMPs). Additionally, this also causes inhibition of DAMP-induced inflammatory cytokine activation. As a result, while CD24 dampens immune activation, it does not affect immune recognition, hence does not interfere with viral clearance. Exosomes are vesicles that play a role in intercellular communication [65]. They have been reported to be useful delivery vehicles, as they can increase stability and extend bioavailability of therapeutic molecules, as evidenced by multiple ongoing clinical trials [66,67]. Additionally, a recent report showed that nebulized exosomes can help repair PF [68]. To combine the benefits of both CD24 and exosomes, the authors developed CD-24 enriched exosomes (EXO-CD24) as a targeted therapy against COVID-19 immune activation. 

The authors initially reported creation of a HEK293 cell line stably transfected with CD24, which they used for purification of exosomes displaying high levels of CD24 (EXO-CD24) [69]. The authors utilized flow cytometry and western blot analysis to demonstrate high expression of CD24 on the isolated exosomes. Further characterization studies showed that EXO-CD24 had a particle size of approximately 100–200 nm. Additionally, EXO-CD24 had a spherical morphology with clearly visible lipid bilayers and vesicular internal structures.

**Figure 6:**
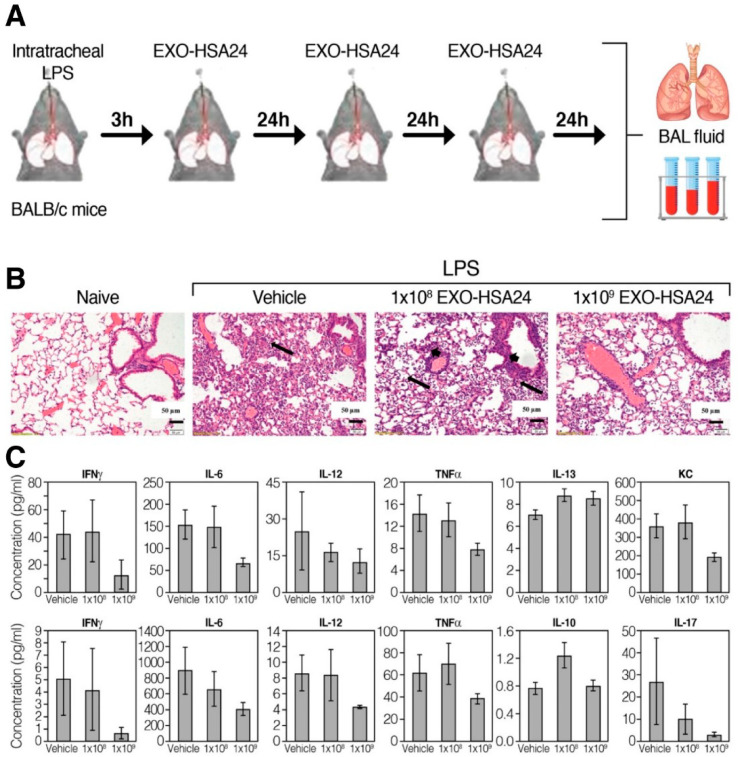
Therapeutic efficacy of EXO-CD24 in mouse ARDS model. (**A**) Study design, which shows mice were challenged using intrathecal administration of LPS followed by EXO-CD24 administration once daily for 3 days. Mice were then sacrificed, and serum and BAL were collected. (**B**) Representative hematoxylin and eosin (H&E) stained histological images of lung tissue showing extensive neutrophil infiltration in saline and low dose-treated mice as opposed to considerable reduction in neutrophil infiltration in high dose-treated mice. (**C**) Levels of cytokines in serum (upper panel) and BAL (lower panel). Reprinted with permission from Shapira S. et al. EMBO Mol Med (2022)14: e15997 [63]. Copyright 2022 EMBO Press.

The authors also demonstrated inhibition of inflammatory cytokine/chemokine secretion in an EXO-CD24-treated human monocyte cell line, U937. In vivo studies with mice were performed to assess the toxicity as well as efficacy. For safety assessment, a dose of either 5 × 10^8^ or 1 × 10^9^ EXO-CD24 was administered by inhalation, once daily, for 5 days (Figure 6A). Animals were either sacrificed on day 6 or followed for an additional week. Saline, the carrier for the exosomes, was used as vehicle. The authors reported that even for the highest dose, no adverse effects or differences were observed between control and treated groups in behavior, food and water consumption, body weight, organ weight at the end of the study, or in hematology, blood chemistry, and urine analyses. Additionally, histological evaluation of organs showed no toxicity concerns. To assess the ability of EXO-CD24 in reducing inflammatory cytokines and lung inflammation, a mouse model of acute respiratory distress syndrome (ARDS) was used (Figure 6B). ARDS-affected mice were administered either 5 × 10^8^ or 1 × 10^9^ EXO-CD24 once daily for 3 days. After 3 days of treatment, the mice were sacrificed and assessed for histological differences as well as levels of various inflammatory markers. The authors reported that the animals treated with the lower dose had moderate-to-severe lung injury, while animals with the higher dose showed a marked reduction in lung injury. Additionally, a significant dose-dependent reduction in cytokines was observed (Figure 6C). Collectively, these findings suggested that EXO-CD24 was a safe and efficacious treatment for COVID-19-induced lung inflammation.

**Figure 7:**
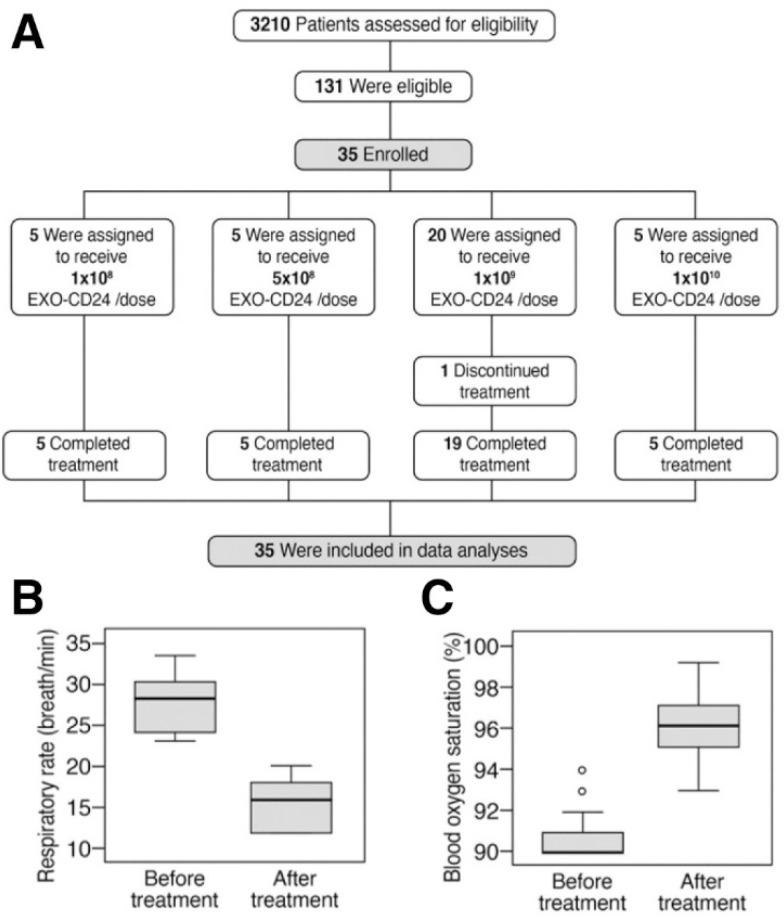
Patient enrollment and clinical results. (**A**) Clinical study design. (**B**) Respiratory rate and (**C**) blood oxygen before and after treatment. (Reprinted with permission from Shapira S. et al. EMBO Mol Med (2022)14: e15997 [63]. Copyright 2022 EMBO Press.

Following promising observations in animal studies, a Phase Ib/IIa clinical study was performed mainly with an intent to assess safety, with an additional focus on pharmacokinetics. Thirty-five patients were enrolled in four dose escalation groups (1 × 10^8^, 5 × 10^8^, 1 × 10^9^, 1 × 10^10^ EXO-CD24/dose) (Figure 7A). An increase in respiratory rate and blood oxygen levels showed the promise of this therapy in treatment of lung injury (Figure 7B,C).

**Figure 8:**
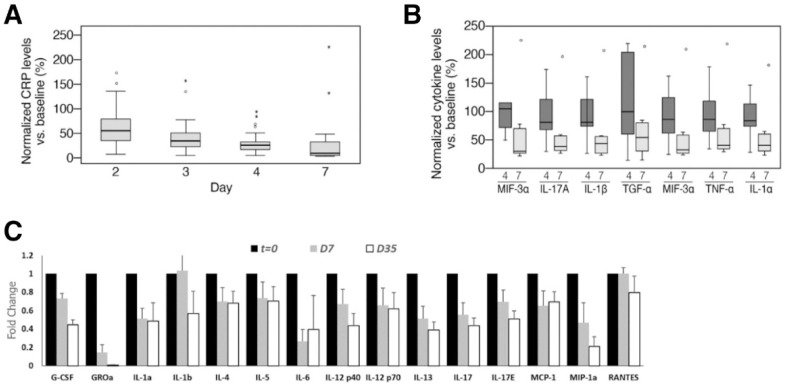
Patients’ systemic inflammatory markers. (**A**) Plot showing systemic CRP values normalized against day 0 values for 35 patients. (**B**) Systemic cytokine levels measured in 8 patients on days 3/4 and days 7/8 after treatment with EXO-CD24. (**C**) Fold change in cytokine levels measured in 24 patients during course of EXO-CD24 treatment. Reprinted with permission from Shapira S. et al. EMBO Mol Med (2022)14: e15997 [63]. Copyright 2022 EMBO Press.

Additionally, in patients that participated in the study, the decrease in serum cytokine arrays and inflammatory markers in a time-dependent manner suggested the efficacy of EXO-CD24 (Figure 8). Overall, the results from this study showed that EXO-CD24 had a high tolerability and potential efficacy that justified further clinical investigations.

Despite these promising results, lack of standardized isolation, characterization, and large-scale production technologies for exosomes remains a major limiting factor in the clinical translation of exosomes. Similarly, the recommended storage temperature for exosomes is −80 °C which introduces additional barriers in their translation. 

### 4.3. Mannosylated Albumin-siRNA NPs

It has been reported that PF involves activation of the innate and adaptive immune pathways that release inflammatory cytokines [70]. During this process, a crucial role is played by resident alveolar macrophages, which are replaced by newly penetrating monocyte-derived macrophages (Mo-AMs) that transition into alveolar macrophages [71]. These newly arriving macrophages have an overexpression of the mannose receptor CD206 [72]. Current therapies such as pirfenidone and nintedanib only dampen immune activation [73]. However, there is no target-specific approach to disease-inducing macrophage population [74].

Singh and co-workers leveraged these mannose receptors by developing mannosylated albumin nanoparticles (MANPs) that could be internalized by CD206+ macrophages [75]. They utilized these nanoparticles to deliver a TGFβ1 siRNA that could reduce inflammatory cytokine secretion and prevent PF [76]. The MANPs were prepared by a coacervation process and coated with D-mannose. Albumin-based NPs have been widely reported to possess safety as well as relevance to clinical translation. The mannose coating was enabled by opening the aldehyde groups at low pH and high temperatures to allow reaction of the activated mannose with free amine groups of ANPs. The surface mannose concentration was optimized at 8 mM, which was the concentration at which the NP surface appeared to be saturated, as evidenced by quantification studies and ^1^NMR. Coating of ANPs with mannose resulted in an almost two-fold increase in particle size, from 60 nm to 100 nm. The cellular uptake of the synthesized MANPs in macrophages was assessed by flow cytometry. Additionally, the authors showed that the uptake was a result of CD206 targeting by incubating these cells with CD206 antibody, which resulted in a reduction in the macrophage uptake. 

To assess the application of MANPs as delivery vehicles, MANPs encapsulating TGFβ1 siRNA were synthesized. The entrapment efficiency of siRNA was determined to be about 60%, and about 50% release of siRNA was observed in 5 days in PBS pH 7.4 at 37 °C. Fluorescently labeled siRNA was encapsulated, and macrophage uptake was assessed using flow cytometry and confocal microscopy. These studies showed that MANP-siRNA had significantly more internalization as compared to free siRNA.

**Figure 9:**
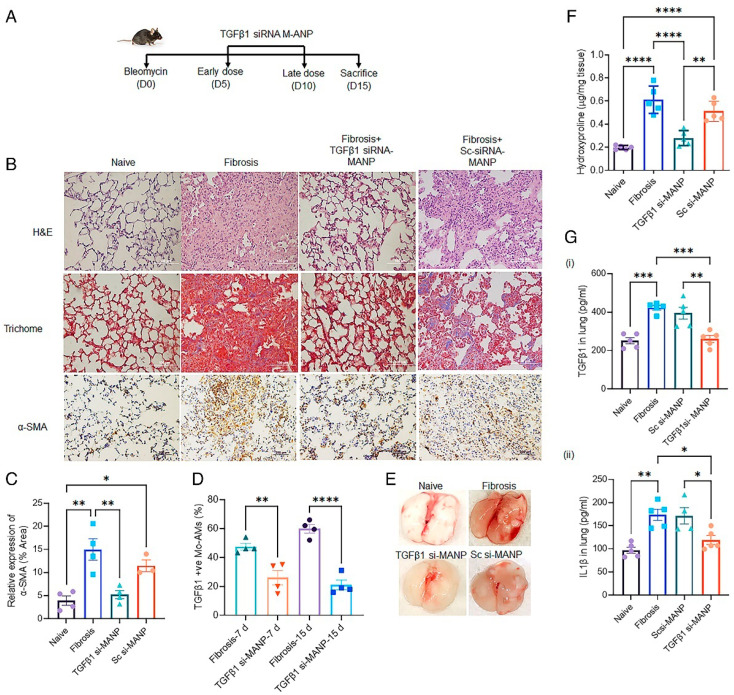
Mitigation of lung fibrosis by MANPs with TGFβ1 siRNA. (**A**) Study design and administration schedule for therapeutic evaluation in mice with bleomycin-induced lung fibrosis. (**B**) Representative histological images showing reduction in lung fibrosis after treatment with TGFβ1siRNA-MANP as compared to untreated mice and mice treated with MANPs incorporating scrambled siRNA. (**C**) Quantification of α-SMA staining. (**D**) Reduction in TGFβ1 expression in Mo-AMs. (**E**) Reduction in fibrotic mass after treatment. (**F**) Reduction in collagen deposition shown by hydroxyproline assay. (**G**) Quantification of profibrotic cytokines (i) TGFβ1 and (ii) IL1β in lungs. * *p* < 0.05, ** *p* < 0.01, *** *p* < 0.001 and **** *p* < 0.0001. Reprinted with permission from Singh A. et al. PNAS (2022) 119 (15) e2121098119 [75]. Copyright 2022 The National Academy of Sciences.

To assess the in vivo efficacy of TGFβ1 siRNA-loaded MANPs, the authors utilized a bleomycin-induced PF model, which was 15 days long (Figure 9A). TGFβ1 siRNA-loaded MANPs were administered on days 5 and 10. On day 15, the mice were sacrificed, and histological assessments were performed (Figure 9B). A marked reduction was observed in the TGFβ1 siRNA-loaded MANP-treated mice. Additionally, a significant reduction in fibrotic mass as well as collagen deposition was observed (Figure 9E). The treated group also showed a three-fold reduction in macrophage proliferation in the lungs (Figure 9F,G). Further assessment was performed to assess the levels of proinflammatory cytokines TGFβ1 and IL-1β in lungs by ELISA. It was observed that the TGFβ1 siRNA-loaded MANP-treated mice showed marked reduction in the cytokine levels. 

Lung function was assessed by measuring multiple mechanical properties of the respiratory system using an instrument called flexiVent. Marked improvements were observed in the lung function of animals treated with TGFβ1 siRNA-loaded MANPs. Overall, the authors demonstrated the therapeutic efficacy of MANPs to target a specific subset of lung macrophages and mitigate PF.

### 4.4. Nanostructured Hydroxychloroquine

**Figure 10:**
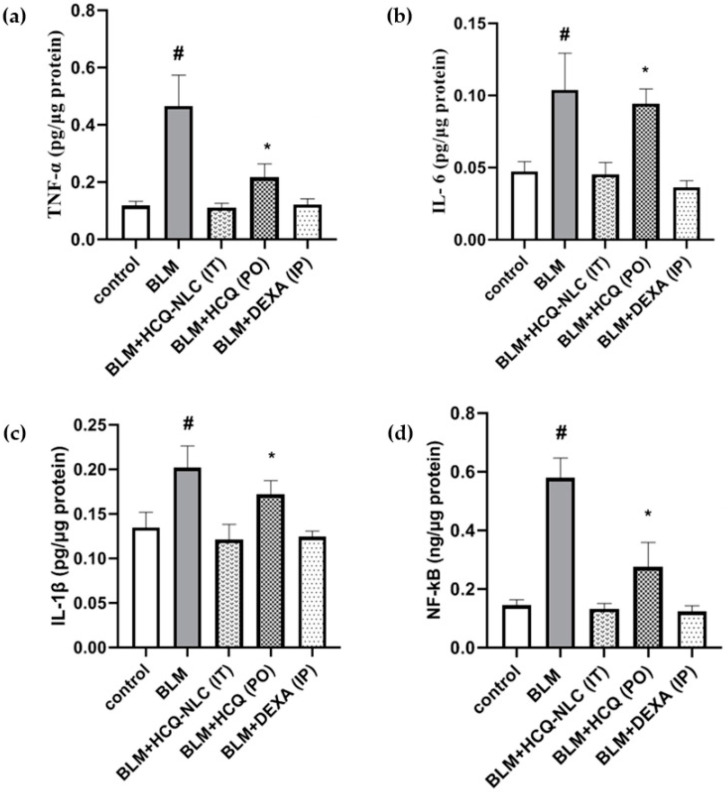
Reduction in pro-inflammatory cytokines (**a**) TNF-α; (**b**) IL-6; (**c**) IL-1β and (**d**) NF-κβ following treatment with various HCQ formulations. # Significant (*p* < 0.05) versus all groups. * Significant (*p* < 0.05) versus the control group and HCQ-NLC group Reprinted with permission from Ali AS. et al. Polymers (Basel). 2022 July; 14(13): 2616 [81]. Copyright 2022 MDPI.

Hydroxychloroquine (HCQ) is an inexpensive drug that has been commonly indicated for the treatment of malaria [77]. Additionally, in recent times, HCQ has been investigated for its anti-inflammatory effects in the treatment of disorders such as rheumatoid arthritis, lupus and inflammatory bowel disease [78]. There have been multiple reports of modifying HCQ to alter its pharmacokinetics so that its therapeutic action can be made more specific [79,80]. Following a similar rationale, Ali and co-workers investigated hydroxychloroquine nanostructured lipid carriers (HCQ-NLCs) as a pulmonary delivery system to treat COVID-19-induced PF [81]. HCQ-NLC was prepared using a hot emulsification-ultrasonication method. The final nanocarrier comprised mainly almond oil and Compritol 888ATO. Additionally, L-phosphatidylcholine was included as an amphiphilic surfactant to promote the stability of NLCs. The total lipid content used in the synthesis was about 10% *w*/*v*, while the surfactant was added at 2% *w*/*v*. Targeted HCQ content was 50 mg. Characterization of the obtained NLCs showed a particle size of approximately 250 nm with a PDI of 0.3. In vitro release testing showed that 15% of HCQ was released in 30 min, followed by a cumulative release of 80% over 12 h. To evaluate in vivo efficacy, the authors investigated HCQ-NLCs in a murine bleomycin (BLM)-induced fibrosis model. Mice with fibrosis were divided into different treatment groups and administered with six doses of HCQ-NLC via intrathecal route, HCQ suspension via oral route, and Dexamethasone (Dexa) administered via intraperitoneal route. Dexa was included as a positive control, as it is used extensively in the management of PF. Post-sacrifice, lung tissue was utilized for analysis of pro-inflammatory cytokine levels and histopathological evaluation. All three treatment groups showed a reduction in TNFα, IL-6, IL-1β and NFK-β as compared to the untreated mice (Figure 10). Additionally, HCQ-NLC-administered mice reduced pro-inflammatory cytokine levels to base levels as compared to the HCQ-treated group. Similar results were observed with the histopathological evaluation. HCQ-NLC-treated mice showed marked open alveoli free of any inflammatory cell infiltration. Slight improvement was observed for HCQ-administered mice. 

This report lacked depth in its study design in terms of explaining why the authors chose to evaluate the efficacy of different treatments via different routes of administration. In our opinion, this approach to study design does not provide adequate comparative information to confirm whether the HCQ-NLCs are significantly better than the other treatment groups. Additionally, intrathecal delivery has low patient compliance, and the possibility of burst release of payload from NLCs leading to adverse effects as well as chances of residual solvent-associated toxicity from the use of organic solvents are major drawbacks of this delivery system. Nevertheless, this report highlights a potential nanotherapeutic approach where an existing small molecule drug was packaged into a nanocarrier and its application to PF was assessed.

### 4.5. PLGA-PEG-G0-C14-IL11 siRNA NPs

Bai and co-workers reported the development of a novel lipid–polymer platform consisting of lipid-like compound G0-C14 and PLGA-PEG [82]. The developed NPs encapsulated siRNA against IL11, which is a proinflammatory cytokine [83]. The developed NPs enabled pulmonary delivery of siRNA through bronchiolar and alveolar epithelium on inhalation. The authors reported that these NPs had good biocompatibility as well as the ability to withstand the shear stress generated during nebulization. In vitro studies were performed to exhibit effective gene silencing. In vivo studies in mice showed that these NPs did not elicit any immune reactions in lung or liver, thus showing their promise as an efficient inhaled RNA therapy.

**Figure 11:**
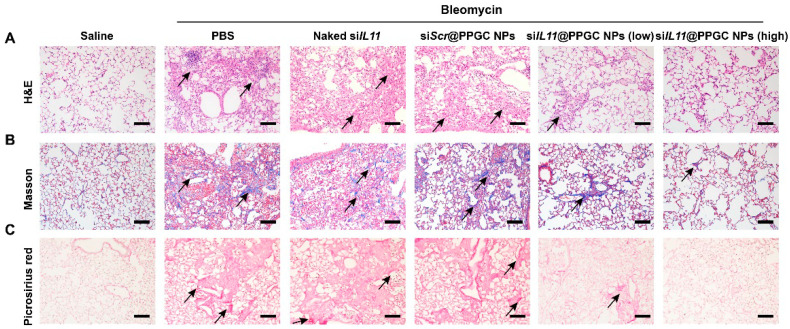
Representative histological lung tissue images showing therapeutic efficacy of siIL11 siRNA NPs in pulmonary fibrosis mouse model. Reprinted with permission from Bai X. et al. Science Advances. 2022 June; 8(25) [82]. Copyright 2022 Science.

Additionally, the authors assessed the therapeutic efficacy of these NPs in a bleomycin-induced PF mouse model, which is clinically relevant and has been widely used for PF studies [84]. NP-treated mice showed a marked reduction in inflammatory signs (Figure 11). Histologically, a significant reduction in thickness of alveolar septa was observed. Additionally, restoration of impaired alveolar barrier and reduced collagen deposition were observed. 

The authors indicate that while promising, the evaluation of this system in other models of PF would greatly help, considering that only one disease model does not cover all characteristics of the disease. Nevertheless, the authors believe that this system can be used for simultaneous delivery of multiple siRNAs, which could potentially provide synergistic benefits. 

## 5. Challenges and Future Perspectives

The COVID-19 pandemic has been a major threat to human health. While the medical and pharmaceutical industry has been quick to develop an initial understanding of this disease and developing vaccines, there has not been much focus on the long-term effects of COVID-19 [85]. Partly, this is because the pandemic is about 3 years old, so long-term effects are still being monitored [86,87]. However, a significant percentage of COVID-19-affected individuals have shown a certain degree of lung damage [88]. In rare cases, a few patients with existing lung conditions have developed PF [89,90,91]. Overall, it remains to be seen whether COVID-19-affected patients exhibit progressive PF in the long term. Although PF is a well characterized disease, COVID-19-induced PF might have a different pathophysiology and consequently its own treatment challenges. The current mainstay of therapies for COVID-19-induced PF are still drugs that are indicated for PF. However, recently there has been a shift in the field to evaluate these therapies specifically for COVID-19-induced PF. As a result, there are multiple clinical trials ongoing to assess the efficacy of PF drugs in COVID-19-induced PF [92]. However, the major challenges include safety as well as specificity of these therapeutics. Most of these drugs function by silencing the immune system [93]. Lack of specificity results in non-specific immune silencing, which can lead to many concerning side effects. Nanotherapeutic approaches have shown benefits over similar concerns in the context of other disease areas [94,95,96]. As a result, there are many reports of evaluations of various nanotherapeutic systems in COVID-19-induced PF [97,98,99]. 

In the future, the hope is that more studies will result in therapies with higher margins of safety and specificity toward COVID-19-induced PF [100,101,102,103]. However, we are still in the preliminary stages of nanomedicine applications for lung diseases. There are several challenges and considerations that must be addressed before these therapeutics can be assimilated into mainstream treatment options [104]. Many reports have discussed the delivery challenges. These challenges are usually either delivery or formulation challenges. However, in relation to the bigger picture, the major challenge to clinical translation would be the development of industrial processes to scale up these therapies to produce at scale large enough to supply phase III clinical trials and commercial production [105]. Additionally, processes capable of consistently producing uniform therapeutics will be a challenge. The development of critical quality attributes as well as reliable methods to assess these attributes will significantly impact the translation of nanotherapeutics. Furthermore, the development of robust and biologically representative animal models will bolster pre-clinical evaluations of these therapeutics. In terms of drug product development, storage, and stability during the shelf-life of these nanomedicines will also be a significant challenge. The advent of COVID-19 vaccines brought significant attention to formulation compositions as well as components of the nano-delivery systems and their influence and behavior under various storage conditions [106]. This has led to several reports that elucidated the impact of storage conditions on the thermal stability of nanotherapeutics. Addressing thermal stability concerns will represent a significant challenge. Better understanding of the regulatory landscape, especially surrounding lung-targeted therapies, will be needed. Overall, we are headed in the right direction in terms of starting to understand lung-targeted therapies and patient needs. However, much more knowledge is still needed to allow for successful bedside translation of these nanotherapies [107].

## Figures and Tables

**Table 1:** Ongoing clinical trials for COVID-19-induced PF therapy.

Clinical Trial ID	Title	Intervention	Sponsor
NCT04607928	Phase-II Randomized Clinical Trial to Evaluate the Effect of Pirfenidone Compared to Placebo in Post-COVID19	Drug: Pirfenidone Drug: Placebo	Institut d’Investigació Biomèdica de Bellvitge
NCT04818489	Impact of Colchicine on the Clinical Outcome of COVID-19 and the Development of Post-COVID-19 Pulmonary Fibrosis: Randomized Controlled Clinical Trial	Drug: Colchicine 0.5 mg Other: the standard protocol only	ClinAmygate
NCT04551781	Short Term Low Dose Corticosteroids for Management of Post Covid-19 Pulmonary Fibrosis	Drug: 20 mg Prednisone for 14 days Drug: control	South Valley University
NCT05648734	Impact of Anti-Inflammatory and Anti-Fibrotic Drugs on Post-acute COVID-19 Pulmonary Fibrosis	Drug: Corticosteroids alone Drug: Corticosteroids + Colchicine Drug: Corticosteroids + Pirfenidone Drug: Corticosteroids + Colchicine + Pirfenidone for ≥14 day	Mansoura University
NCT04279197	Efficacy and Safety of Fuzheng Huayu Tablets in Post-COVID-19 Patients with Pulmonary Inflammation and Fibrosis: A Multicenter Double-blind Randomized Controlled Trial	Drug: Fuzheng Huayu Tablet Drug: Vitamin C tablets Drug: Placebo Other: respiratory function rehabilitation training	ShuGuang Hospital
NCT04541680	Nintedanib for the Treatment of SARS-Cov-2 Induced Pulmonary Fibrosis	Drug: Nintedanib 150 mg Other: Placebo	Assistance Publique–Hôpitaux de Paris
NCT04856111	A Study of the Efficacy and Safety of Pirfenidone vs. Nintedanib in the Treatment of Fibrotic Lung Disease After Coronavirus Disease-19	Drug: Pirfenidone Drug: Nintedanib	Postgraduate Institute of Medical Education and Research
NCT04948203	SECOVID: A Multi-center, Randomized, Dose-ranging Parallel-group Trial Assessing the Efficacy of Sirolimus in Hospitalized Patients With COVID-19 Pneumonia for the Prevention of Post-COVID Fibrosis	Drug: Sirolimus	University of Chicago
NCT05387239	Safety and Effectiveness of EV-Pure + WJ-Pure Treatment on Pulmonary Fibrosis Secondary to Covid-19	Drug: EV-Pure™ and WJ-Pure™ plus standard care Drug: Placebo (Saline plus standard care)	Vitti Labs, LLC
NCT04645368	Multicenter, Open-label Prospective Cohort Study of the Efficacy and Safety of the Inclusion of Longidaze in the Prevention and Treatment of Post-inflammatory Pulmonary Fibrosis and Interstitial Lung Diseases Caused by COVID-19	Drug: bovhyaluronidase azoxymer	NPO Petrovax
NCT04805086	Phase I/II MONACO Cell Therapy Study: Monocytes as an Anti-fibrotic Treatment After COVID-19	Biological: MON002	Guy’s and St Thomas’ NHS Foundation Trust
NCT04912011	The Use of a Mineralocorticoid Receptor Antagonist (Spironolactone) in the Treatment of Pulmonary Fibrosis Associated With SARS-CoV-2 Infection	Drug: Canrenoate Potassium Drug: Normal Saline	Pomeranian Medical University Szczecin
NCT04338802	Efficacy and Safety of Nintedanib Ethanesulfonate Soft Capsule in the Treatment of Pulmonary Fibrosis in Patients with Moderate to Severe COVID-9 (COVID 19): a Single-center, Randomized, Placebo-controlled Study	Drug: Nintedanib 150 mg Other: Placebo	Tongji Hospital
NCT04619680	Early Nintedanib Deployment in COVID-19 Interstitial Lung Disease	Drug: Nintedanib Drug: Placebo	Icahn School of Medicine at Mount Sinai
NCT04482595	A Phase 2 Study of BIO 300 Oral Suspension in Discharged COVID-19 Patients	Drug: BIO 300 Oral Suspension Drug: Placebo	Humanetics Corporation
NCT04537130	Phase Ib Controlled Exploratory Trial for Treatment of Fibrosing Interstitial Lung Disease Patients Secondary to SARS-CoV-2 Infection with IN01 Vaccine (COVINVAC)	Biological: IN01 vaccine	Instituto Oncológico

## Data Availability

Not applicable.

## References

[1] George P.M., Patterson C.M., Reed A.K., Thillai M. (2019). Lung Transplantation for Idiopathic Pulmonary Fibrosis. Lancet Respir. Med..

[2] Meyer K.C. (2017). Pulmonary Fibrosis, Part I: Epidemiology, Pathogenesis, and Diagnosis. Expert Rev. Respir. Med..

[3] King T.E., Pardo A., Selman M. (2011). Idiopathic Pulmonary Fibrosis. Lancet.

[4] Travis W.D., Costabel U., Hansell D.M., King T.E., Lynch D.A., Nicholson A.G., Ryerson C.J., Ryu J.H., Selman M., Wells A.U. (2013). An Official American Thoracic Society/European Respiratory Society Statement: Update of the International Multidisciplinary Classification of the Idiopathic Interstitial Pneumonias. Am. J. Respir. Crit. Care Med..

[5] Seibold M.A., Wise A.L., Speer M.C., Steele M.P., Brown K.K., Loyd J.E., Fingerlin T.E., Zhang W., Gudmundsson G., Groshong S.D. (2011). A Common MUC5B Promoter Polymorphism and Pulmonary Fibrosis. N. Engl. J. Med..

[6] Newton C.A., Batra K., Torrealba J., Kozlitina J., Glazer C.S., Aravena C., Meyer K., Raghu G., Collard H.R., Garcia C.K. (2016). Telomere-Related Lung Fibrosis Is Diagnostically Heterogeneous but Uniformly Progressive. Eur. Respir. J..

[7] Yang I. (2012). V Epigenomics of Idiopathic Pulmonary Fibrosis. Epigenomics.

[8] Tzouvelekis A., Kaminski N. (2015). Epigenetics in Idiopathic Pulmonary Fibrosis. Biochem. Cell Biol..

[9] Caminati A., Madotto F., Cesana G., Conti S., Harari S. (2015). Epidemiological Studies in Idiopathic Pulmonary Fibrosis: Pitfalls in Methodologies and Data Interpretation. Eur. Respir. Rev..

[10] Raghu G., Chen S.-Y., Yeh W.-S., Maroni B., Li Q., Lee Y.-C., Collard H.R. (2014). Idiopathic Pulmonary Fibrosis in US Medicare Beneficiaries Aged 65 Years and Older: Incidence, Prevalence, and Survival, 2001–11. Lancet Respir. Med..

[11] Tanni S.E., Fabro A.T., de Albuquerque A., Ferreira E.V.M., Verrastro C.G.Y., Sawamura M.V.Y., Ribeiro S.M., Baldi B.G. (2021). Pulmonary Fibrosis Secondary to COVID-19: A Narrative Review. Expert Rev. Respir. Med..

[12] Zou J.-N., Sun L., Wang B.-R., Zou Y., Xu S., Ding Y.-J., Shen L.-J., Huang W.-C., Jiang X.-J., Chen S.-M. (2021). The Characteristics and Evolution of Pulmonary Fibrosis in COVID-19 Patients as Assessed by AI-Assisted Chest HRCT. PLoS ONE.

[13] Fisher M., Nathan S.D., Hill C., Marshall J., Dejonckheere F., Thuresson P.-O., Maher T.M. (2017). Predicting Life Expectancy for Pirfenidone in Idiopathic Pulmonary Fibrosis. J. Manag. Care Spec. Pharm..

[14] Richeldi L., du Bois R.M., Raghu G., Azuma A., Brown K.K., Costabel U., Cottin V., Flaherty K.R., Hansell D.M., Inoue Y. (2014). Efficacy and Safety of Nintedanib in Idiopathic Pulmonary Fibrosis. N. Engl. J. Med..

[15] King T.E., Bradford W.Z., Castro-Bernardini S., Fagan E.A., Glaspole I., Glassberg M.K., Gorina E., Hopkins P.M., Kardatzke D., Lancaster L. (2014). A Phase 3 Trial of Pirfenidone in Patients with Idiopathic Pulmonary Fibrosis. N. Engl. J. Med..

[16] Fadista J., Kraven L.M., Karjalainen J., Andrews S.J., Geller F., Baillie J.K., Wain L.V., Jenkins R.G., Feenstra B. (2021). Shared Genetic Etiology between Idiopathic Pulmonary Fibrosis and COVID-19 Severity. eBioMedicine.

[17] Wendisch D., Dietrich O., Mari T., von Stillfried S., Ibarra I.L., Mittermaier M., Mache C., Chua R.L., Knoll R., Timm S. (2021). SARS-CoV-2 Infection Triggers Profibrotic Macrophage Responses and Lung Fibrosis. Cell.

[18] Wiersinga W.J., Rhodes A., Cheng A.C., Peacock S.J., Prescott H.C. (2020). Pathophysiology, Transmission, Diagnosis, and Treatment of Coronavirus Disease 2019 (COVID-19): A Review. JAMA.

[19] Parimon T., Espindola M., Marchevsky A., Rampolla R., Chen P., Hogaboam C.M. (2022). Potential Mechanisms for Lung Fibrosis Associated with COVID-19 Infection. QJM An Int. J. Med..

[20] Rendeiro A.F., Ravichandran H., Bram Y., Chandar V., Kim J., Meydan C., Park J., Foox J., Hether T., Warren S. (2021). The Spatial Landscape of Lung Pathology during COVID-19 Progression. Nature.

[21] Flaifel A., Kwok B., Ko J., Chang S., Smith D., Zhou F., Chiriboga L.A., Zeck B., Theise N., Rudym D. (2022). Pulmonary Pathology of End-Stage COVID-19 Disease in Explanted Lungs and Outcomes After Lung Transplantation. Am. J. Clin. Pathol..

[22] Cheresh P., Kim S.-J., Tulasiram S., Kamp D.W. (2013). Oxidative Stress and Pulmonary Fibrosis. Biochim. Biophys. Acta—Mol. Basis Dis..

[23] Tran S., Ksajikian A., Overbey J., Li P., Li Y. (2022). Pathophysiology of Pulmonary Fibrosis in the Context of COVID-19 and Implications for Treatment: A Narrative Review. Cells.

[24] Sehlmeyer K., Ruwisch J., Roldan N., Lopez-Rodriguez E. (2020). Corrigendum: Alveolar Dynamics and Beyond—The Importance of Surfactant Protein C and Cholesterol in Lung Homeostasis and Fibrosis. Front. Physiol..

[25] Seibold M.A., Smith R.W., Urbanek C., Groshong S.D., Cosgrove G.P., Brown K.K., Schwarz M.I., Schwartz D.A., Reynolds S.D. (2013). The Idiopathic Pulmonary Fibrosis Honeycomb Cyst Contains A Mucocilary Pseudostratified Epithelium. PLoS ONE.

[26] Conforti F., Ridley R., Brereton C., Alzetani A., Johnson B., Marshall B.G., Fletcher S.V., Ottensmeier C.H., Richeldi L., Skipp P. (2020). Paracrine SPARC Signaling Dysregulates Alveolar Epithelial Barrier Integrity and Function in Lung Fibrosis. Cell Death Discov..

[27] Evans M.J., Van Winkle L.S., Fanucchi M.V., Plopper C.G. (1999). The Attenuated Fibroblast Sheath of the Respiratory Tract Epithelial–Mesenchymal Trophic Unit. Am. J. Respir. Cell Mol. Biol..

[28] Ghazavi A., Ganji A., Keshavarzian N., Rabiemajd S., Mosayebi G. (2021). Cytokine Profile and Disease Severity in Patients with COVID-19. Cytokine.

[29] Colarusso C., Maglio A., Terlizzi M., Vitale C., Molino A., Pinto A., Vatrella A., Sorrentino R. (2021). Post-COVID-19 Patients Who Develop Lung Fibrotic-like Changes Have Lower Circulating Levels of IFN-β but Higher Levels of IL-1α and TGF-β. Biomedicines.

[30] Melms J.C., Biermann J., Huang H., Wang Y., Nair A., Tagore S., Katsyv I., Rendeiro A.F., Amin A.D., Schapiro D. (2021). A Molecular Single-Cell Lung Atlas of Lethal COVID-19. Nature.

[31] Yao C., Bora S.A., Parimon T., Zaman T., Friedman O.A., Palatinus J.A., Surapaneni N.S., Matusov Y.P., Cerro Chiang G., Kassar A.G. (2021). Cell-Type-Specific Immune Dysregulation in Severely Ill COVID-19 Patients. Cell Rep..

[32] Bharat A., Querrey M., Markov N.S., Kim S., Kurihara C., Garza-Castillon R., Manerikar A., Shilatifard A., Tomic R., Politanska Y. (2020). Lung Transplantation for Patients with Severe COVID-19. Sci. Transl. Med..

[33] Aesif S.W., Bribriesco A.C., Yadav R., Nugent S.L., Zubkus D., Tan C.D., Mehta A.C., Mukhopadhyay S. (2021). Pulmonary Pathology of COVID-19 Following 8 Weeks to 4 Months of Severe Disease: A Report of Three Cases, Including One With Bilateral Lung Transplantation. Am. J. Clin. Pathol..

[34] Horowitz J.C., Thannickal V.J. (2006). Epithelial-Mesenchymal Interactions in Pulmonary Fibrosis. Semin. Respir. Crit. Care Med..

[35] Maher T.M., Wells A.U., Laurent G.J. (2007). Idiopathic Pulmonary Fibrosis: Multiple Causes and Multiple Mechanisms?. Eur. Respir. J..

[36] Uhal B.D., Joshi I., Hughes W.F., Ramos C., Pardo A., Selman M. (1998). Alveolar Epithelial Cell Death Adjacent to Underlying Myofibroblasts in Advanced Fibrotic Human Lung. Am. J. Physiol. Cell. Mol. Physiol..

[37] Yao L., Conforti F., Hill C., Bell J., Drawater L., Li J., Liu D., Xiong H., Alzetani A., Chee S.J. (2019). Paracrine Signalling during ZEB1-Mediated Epithelial–Mesenchymal Transition Augments Local Myofibroblast Differentiation in Lung Fibrosis. Cell Death Differ..

[38] Jiang P., Gil de Rubio R., Hrycaj S.M., Gurczynski S.J., Riemondy K.A., Moore B.B., Omary M.B., Ridge K.M., Zemans R.L. (2020). Ineffectual Type 2–to–Type 1 Alveolar Epithelial Cell Differentiation in Idiopathic Pulmonary Fibrosis: Persistence of the KRT8hi Transitional State. Am. J. Respir. Crit. Care Med..

[39] Cassandras M., Wang C., Kathiriya J., Tsukui T., Matatia P., Matthay M., Wolters P., Molofsky A., Sheppard D., Chapman H. (2020). Gli1+ Mesenchymal Stromal Cells Form a Pathological Niche to Promote Airway Progenitor Metaplasia in the Fibrotic Lung. Nat. Cell Biol..

[40] Prasse A., Binder H., Schupp J.C., Kayser G., Bargagli E., Jaeger B., Hess M., Rittinghausen S., Vuga L., Lynn H. (2018). BAL Cell Gene Expression Is Indicative of Outcome and Airway Basal Cell Involvement in Idiopathic Pulmonary Fibrosis. Am. J. Respir. Crit. Care Med..

[41] Blackwell T.S., Tager A.M., Borok Z., Moore B.B., Schwartz D.A., Anstrom K.J., Bar-Joseph Z., Bitterman P., Blackburn M.R., Bradford W. (2013). Future Directions in Idiopathic Pulmonary Fibrosis Research. An NHLBI Workshop Report. Am. J. Respir. Crit. Care Med..

[42] Koli K., Myllärniemi M., Vuorinen K., Salmenkivi K., Ryynänen M.J., Kinnula V.L., Keski-Oja J. (2006). Bone Morphogenetic Protein-4 Inhibitor Gremlin Is Overexpressed in Idiopathic Pulmonary Fibrosis. Am. J. Pathol..

[43] Keski-Oja J., Koli K., Lohi J., Laiho M. (1991). Growth Factors in the Regulation of Plasminogen-Plasmin System in Tumor Cells. Semin. Thromb. Hemost..

[44] Raghow R., Postlethwaite A.E., Keski-Oja J., Moses H.L., Kang A.H. (1987). Transforming Growth Factor-Beta Increases Steady State Levels of Type I Procollagen and Fibronectin Messenger RNAs Posttranscriptionally in Cultured Human Dermal Fibroblasts. J. Clin. Investig..

[45] Shannon J.M., Hyatt B.A. (2004). Epithelial-Mesenchymal Interactions in the Developing Lung. Annu. Rev. Physiol..

[46] Meyer K.C. (2017). Pulmonary Fibrosis, Part II: State-of-the-Art Patient Management. Expert Rev. Respir. Med..

[47] Kreuter M. (2014). Pirfenidone: An Update on Clinical Trial Data and Insights from Everyday Practice. Eur. Respir. Rev..

[48] Taniguchi H., Ebina M., Kondoh Y., Ogura T., Azuma A., Suga M., Taguchi Y., Takahashi H., Nakata K., Sato A. (2010). Pirfenidone in Idiopathic Pulmonary Fibrosis. Eur. Respir. J..

[49] Noble P.W., Albera C., Bradford W.Z., Costabel U., Glassberg M.K., Kardatzke D., King T.E., Lancaster L., Sahn S.A., Szwarcberg J. (2011). Pirfenidone in Patients with Idiopathic Pulmonary Fibrosis (CAPACITY): Two Randomised Trials. Lancet.

[50] Spagnolo P., Maher T.M., Richeldi L. (2015). Idiopathic Pulmonary Fibrosis: Recent Advances on Pharmacological Therapy. Pharmacol. Ther..

[51] King C.S., Nathan S.D. (2015). Practical Considerations in the Pharmacologic Treatment of Idiopathic Pulmonary Fibrosis. Curr. Opin. Pulm. Med..

[52] Wani A., Savithra G.H.L., Abyad A., Kanvinde S., Li J., Brock S., Oupický D. (2017). Surface PEGylation of Mesoporous Silica Nanorods (MSNR): Effect on Loading, Release, and Delivery of Mitoxantrone in Hypoxic Cancer Cells. Sci. Rep..

[53] Roggers R., Kanvinde S., Boonsith S., Oupický D. (2014). The Practicality of Mesoporous Silica Nanoparticles as Drug Delivery Devices and Progress toward This Goal. AAPS PharmSciTech.

[54] Zhu Y., Li J., Kanvinde S., Lin Z., Hazeldine S., Singh R.K., Oupický D. (2015). Self-Immolative Polycations as Gene Delivery Vectors and Prodrugs Targeting Polyamine Metabolism in Cancer. Mol. Pharm..

[55] Deodhar S., Sillman B., Bade A.N., Avedissian S.N., Podany A.T., McMillan J.M., Gautam N., Hanson B., Dyavar Shetty B.L., Szlachetka A. (2022). Transformation of Dolutegravir into an Ultra-Long-Acting Parenteral Prodrug Formulation. Nat. Commun..

[56] Moss D.M., Siccardi M. (2014). Optimizing Nanomedicine Pharmacokinetics Using Physiologically Based Pharmacokinetics Modelling. Br. J. Pharmacol..

[57] Gautam N., McMillan J.M., Kumar D., Bade A.N., Pan Q., Kulkarni T.A., Li W., Sillman B., Smith N.A., Shetty B.L.D. (2021). Lipophilic Nanocrystal Prodrug-Release Defines the Extended Pharmacokinetic Profiles of a Year-Long Cabotegravir. Nat. Commun..

[58] Kulkarni T.A., Bade A.N., Sillman B., Shetty B.L.D., Wojtkiewicz M.S., Gautam N., Hilaire J.R., Sravanam S., Szlachetka A., Lamberty B.G. (2020). A Year-Long Extended Release Nanoformulated Cabotegravir Prodrug. Nat. Mater..

[59] Beyerstedt S., Casaro E.B., Rangel É.B. (2021). COVID-19: Angiotensin-Converting Enzyme 2 (ACE2) Expression and Tissue Susceptibility to SARS-CoV-2 Infection. Eur. J. Clin. Microbiol. Infect. Dis..

[60] Ni W., Yang X., Yang D., Bao J., Li R., Xiao Y., Hou C., Wang H., Liu J., Yang D. (2020). Role of Angiotensin-Converting Enzyme 2 (ACE2) in COVID-19. Crit. Care.

[61] Yang J., Petitjean S.J.L., Koehler M., Zhang Q., Dumitru A.C., Chen W., Derclaye S., Vincent S.P., Soumillion P., Alsteens D. (2020). Molecular Interaction and Inhibition of SARS-CoV-2 Binding to the ACE2 Receptor. Nat. Commun..

[62] Li Z., Wang Z., Dinh P.-U.C., Zhu D., Popowski K.D., Lutz H., Hu S., Lewis M.G., Cook A., Andersen H. (2021). Cell-Mimicking Nanodecoys Neutralize SARS-CoV-2 and Mitigate Lung Injury in a Non-Human Primate Model of COVID-19. Nat. Nanotechnol..

[63] Shapira S., Ben Shimon M., Hay-Levi M., Shenberg G., Choshen G., Bannon L., Tepper M., Kazanov D., Seni J., Lev-Ari S. (2022). A Novel Platform for Attenuating Immune Hyperactivity Using EXO-CD24 in COVID-19 and Beyond. EMBO Mol. Med..

[64] Fang X., Zheng P., Tang J., Liu Y. (2010). CD24: From A to Z. Cell. Mol. Immunol..

[65] Gurung S., Perocheau D., Touramanidou L., Baruteau J. (2021). The Exosome Journey: From Biogenesis to Uptake and Intracellular Signalling. Cell Commun. Signal..

[66] Chen H., Wang L., Zeng X., Schwarz H., Nanda H.S., Peng X., Zhou Y. (2021). Exosomes, a New Star for Targeted Delivery. Front. Cell Dev. Biol..

[67] Herrmann I.K., Wood M.J.A., Fuhrmann G. (2021). Extracellular Vesicles as a Next-Generation Drug Delivery Platform. Nat. Nanotechnol..

[68] Dinh P.-U.C., Paudel D., Brochu H., Popowski K.D., Gracieux M.C., Cores J., Huang K., Hensley M.T., Harrell E., Vandergriff A.C. (2020). Inhalation of Lung Spheroid Cell Secretome and Exosomes Promotes Lung Repair in Pulmonary Fibrosis. Nat. Commun..

[69] Shapira S., Kazanov D., Weisblatt S., Starr A., Arber N., Kraus S. (2011). The CD24 Protein Inducible Expression System Is an Ideal Tool to Explore the Potential of CD24 as an Oncogene and a Target for Immunotherapy in Vitro and in Vivo. J. Biol. Chem..

[70] Wick G., Backovic A., Rabensteiner E., Plank N., Schwentner C., Sgonc R. (2010). The Immunology of Fibrosis: Innate and Adaptive Responses. Trends Immunol..

[71] Zhang L., Wang Y., Wu G., Xiong W., Gu W., Wang C.-Y. (2018). Macrophages: Friend or Foe in Idiopathic Pulmonary Fibrosis?. Respir. Res..

[72] Tsuchiya K., Suzuki Y., Yoshimura K., Yasui H., Karayama M., Hozumi H., Furuhashi K., Enomoto N., Fujisawa T., Nakamura Y. (2019). Macrophage Mannose Receptor CD206 Predicts Prognosis in Community-Acquired Pneumonia. Sci. Rep..

[73] Roach K.M., Castells E., Dixon K., Mason S., Elliott G., Marshall H., Poblocka M.A., Macip S., Richardson M., Khalfaoui L. (2021). Evaluation of Pirfenidone and Nintedanib in a Human Lung Model of Fibrogenesis. Front. Pharmacol..

[74] Ardura J.A., Rackov G., Izquierdo E., Alonso V., Gortazar A.R., Escribese M.M. (2019). Targeting Macrophages: Friends or Foes in Disease?. Front. Pharmacol..

[75] Singh A., Chakraborty S., Wong S.W., Hefner N.A., Stuart A., Qadir A.S., Mukhopadhyay A., Bachmaier K., Shin J.-W., Rehman J. (2022). Nanoparticle Targeting of de Novo Profibrotic Macrophages Mitigates Lung Fibrosis. Proc. Natl. Acad. Sci. USA.

[76] Patel M., Shahjin F., Cohen J.D., Hasan M., Machhi J., Chugh H., Singh S., Das S., Kulkarni T.A., Herskovitz J. (2021). The Immunopathobiology of SARS-CoV-2 Infection. FEMS Microbiol. Rev..

[77] Fox R.I. (1993). Mechanism of Action of Hydroxychloroquine as an Antirheumatic Drug. Semin. Arthritis Rheum..

[78] Chhonker Y.S., Kanvinde S., Ahmad R., Singh A.B., Oupický D., Murry D.J. (2021). Simultaneous Quantitation of Lipid Biomarkers for Inflammatory Bowel Disease Using LC–MS/MS. Metabolites.

[79] Kesharwani S.S., Ahmad R., Bakkari M.A., Rajput M.K.S., Dachineni R., Valiveti C.K., Kapur S., Jayarama Bhat G., Singh A.B., Tummala H. (2018). Site-Directed Non-Covalent Polymer-Drug Complexes for Inflammatory Bowel Disease (IBD): Formulation Development, Characterization and Pharmacological Evaluation. J. Control. Release.

[80] Kanvinde S., Chhonker Y.S., Ahmad R., Yu F., Sleightholm R., Tang W., Jaramillo L., Chen Y., Sheinin Y., Li J. (2018). Pharmacokinetics and Efficacy of Orally Administered Polymeric Chloroquine as Macromolecular Drug in the Treatment of Inflammatory Bowel Disease. Acta Biomater..

[81] Ali A.S., Alrashedi M.G., Ahmed O.A., Ibrahim I.M. (2022). Pulmonary Delivery of Hydroxychloroquine Nanostructured Lipid Carrier as a Potential Treatment of COVID-19. Polymers (Basel).

[82] Bai X., Zhao G., Chen Q., Li Z., Gao M., Ho W., Xu X., Zhang X.-Q. (2023). Inhaled SiRNA Nanoparticles Targeting IL11 Inhibit Lung Fibrosis and Improve Pulmonary Function Post-Bleomycin Challenge. Sci. Adv..

[83] Fung K.Y., Louis C., Metcalfe R.D., Kosasih C.C., Wicks I.P., Griffin M.D.W., Putoczki T.L. (2022). Emerging Roles for IL-11 in Inflammatory Diseases. Cytokine.

[84] Walters D.M., Kleeberger S.R. (2008). Mouse Models of Bleomycin-Induced Pulmonary Fibrosis. Curr. Protoc. Pharmacol..

[85] Raveendran A.V., Jayadevan R., Sashidharan S. (2021). Long COVID: An Overview. Diabetes Metab. Syndr. Clin. Res. Rev..

[86] Lopez-Leon S., Wegman-Ostrosky T., Perelman C., Sepulveda R., Rebolledo P.A., Cuapio A., Villapol S. (2021). More than 50 Long-Term Effects of COVID-19: A Systematic Review and Meta-Analysis. Sci. Rep..

[87] Levine R.L. (2022). Addressing the Long-Term Effects of COVID-19. JAMA.

[88] Suran M. (2021). Autopsies Reveal Lung Damage Patterns From COVID-19. JAMA.

[89] McGroder C.F., Zhang D., Choudhury M.A., Salvatore M.M., D’Souza B.M., Hoffman E.A., Wei Y., Baldwin M.R., Garcia C.K. (2021). Pulmonary Fibrosis 4 Months after COVID-19 Is Associated with Severity of Illness and Blood Leucocyte Telomere Length. Thorax.

[90] Bazdyrev E., Rusina P., Panova M., Novikov F., Grishagin I., Nebolsin V. (2021). Lung Fibrosis after COVID-19: Treatment Prospects. Pharmaceuticals.

[91] Hama Amin B.J., Kakamad F.H., Ahmed G.S., Ahmed S.F., Abdulla B.A., Mohammed S.H., Mikael T.M., Salih R.Q., Ali R.K., Salh A.M. (2022). Post COVID-19 Pulmonary Fibrosis; a Meta-Analysis Study. Ann. Med. Surg..

[92] Mohammadi A., Balan I., Yadav S., Matos W.F., Kharawala A., Gaddam M., Sarabia N., Koneru S.C., Suddapalli S.K., Marzban S. (2022). Post-COVID-19 Pulmonary Fibrosis. Cureus.

[93] Wynn T.A. (2011). Integrating Mechanisms of Pulmonary Fibrosis. J. Exp. Med..

[94] Kanvinde S., Kulkarni T., Deodhar S., Bhattacharya D., Dasgupta A. (2022). Non-Viral Vectors for Delivery of Nucleic Acid Therapies for Cancer. BioTech.

[95] Soares S., Sousa J., Pais A., Vitorino C. (2018). Nanomedicine: Principles, Properties, and Regulatory Issues. Front. Chem..

[96] Ren J., Cai R., Wang J., Daniyal M., Baimanov D., Liu Y., Yin D., Liu Y., Miao Q., Zhao Y. (2019). Precision Nanomedicine Development Based on Specific Opsonization of Human Cancer Patient-Personalized Protein Coronas. Nano Lett..

[97] Velino C., Carella F., Adamiano A., Sanguinetti M., Vitali A., Catalucci D., Bugli F., Iafisco M. (2019). Nanomedicine Approaches for the Pulmonary Treatment of Cystic Fibrosis. Front. Bioeng. Biotechnol..

[98] Skibba M., Drelich A., Poellmann M., Hong S., Brasier A.R. (2020). Nanoapproaches to Modifying Epigenetics of Epithelial Mesenchymal Transition for Treatment of Pulmonary Fibrosis. Front. Pharmacol..

[99] Loo C.-Y., Lee W.-H. (2022). Nanotechnology-Based Therapeutics for Targeting Inflammatory Lung Diseases. Nanomedicine.

[100] White E.S., Thomas M., Stowasser S., Tetzlaff K. (2022). Challenges for Clinical Drug Development in Pulmonary Fibrosis. Front. Pharmacol..

[101] Kaner R.J., Bajwa E.K., El-Amine M., Gorina E., Gupta R., Lazarus H.M., Luckhardt T.R., Mouded M., Posada K., Richeldi L. (2019). Design of Idiopathic Pulmonary Fibrosis Clinical Trials in the Era of Approved Therapies. Am. J. Respir. Crit. Care Med..

[102] Harari S., Caminati A. (2015). Idiopathic Pulmonary Fibrosis: From Clinical Trials to Real-Life Experiences. Eur. Respir. Rev..

[103] Lancaster L., Crestani B., Hernandez P., Inoue Y., Wachtlin D., Loaiza L., Quaresma M., Stowasser S., Richeldi L. (2019). Safety and Survival Data in Patients with Idiopathic Pulmonary Fibrosis Treated with Nintedanib: Pooled Data from Six Clinical Trials. BMJ Open Respir. Res..

[104] Jogdeo C.M., Panja S., Kanvinde S., Kapoor E., Siddhanta K., Oupický D. (2023). Advances in Lipid-Based Codelivery Systems for Cancer and Inflammatory Diseases. Adv. Healthc. Mater..

[105] Hua S., de Matos M.B.C., Metselaar J.M., Storm G. (2018). Current Trends and Challenges in the Clinical Translation of Nanoparticulate Nanomedicines: Pathways for Translational Development and Commercialization. Front. Pharmacol..

[106] Kumar R., Srivastava V., Baindara P., Ahmad A. (2022). Thermostable Vaccines: An Innovative Concept in Vaccine Development. Expert Rev. Vaccines.

[107] Đorđević S., Gonzalez M.M., Conejos-Sánchez I., Carreira B., Pozzi S., Acúrcio R.C., Satchi-Fainaro R., Florindo H.F., Vicent M.J. (2022). Current Hurdles to the Translation of Nanomedicines from Bench to the Clinic. Drug Deliv. Transl. Res..

